# Apes reciprocate food positively and negatively

**DOI:** 10.1098/rspb.2022.2541

**Published:** 2023-05-10

**Authors:** Avi Benozio, Bailey R. House, Michael Tomasello

**Affiliations:** ^1^ Department of Psychology, Hebrew University of Jerusalem, Jerusalem 91905, Israel; ^2^ Department of Psychology, University of York, York YO10 5DD, UK; ^3^ Department of Psychology and Neuroscience, Duke University, Durham, NC 27708, USA; ^4^ Department of Developmental and Comparative Psychology, Max Planck Institute for Evolutionary Anthropology, 04103 Leipzig, Germany

**Keywords:** chimpanzees, reciprocity, cooperation, prosociality

## Abstract

Reciprocal food exchange is widespread in human societies but not among great apes, who may view food mainly as a target for competition. Understanding the similarities and differences between great apes' and humans’ willingness to exchange food is important for our models regarding the origins of uniquely human forms of cooperation. Here, we demonstrate in-kind food exchanges in experimental settings with great apes for the first time. The initial sample consisted of 13 chimpanzees and 5 bonobos in the control phases, and the test phases included 10 chimpanzees and 2 bonobos, compared with a sample of 48 human children aged 4 years. First, we replicated prior findings showing no spontaneous food exchanges in great apes. Second, we discovered that when apes believe that conspecifics have ‘intentionally’ transferred food to them, positive reciprocal food exchanges (food-for-food) are not only possible but reach the same levels as in young children (approx. 75–80%). Third, we found that great apes engage in negative reciprocal food exchanges (no-food for no-food) but to a lower extent than children. This provides evidence for reciprocal food exchange in great apes in experimental settings and suggests that while a potential mechanism of *fostering* cooperation (via positive reciprocal exchanges) may be shared across species, a stabilizing mechanism (via negative reciprocity) is not.

## 1. Introduction

Many aspects of human society are built on reciprocity [1,2], and the practice of food exchange has perhaps been of particular importance in human evolution [3]. In contemporary small-scale, traditional societies, the reciprocal exchange of food is ubiquitous, voluntary and helps to form and cement many social relationships, with rituals often built around feasting and shared food [4–6]. The practice of food exchange could be motivated by various mechanisms, such as unconditional altruism or reciprocity, based on either long-term correlations in behaviour or short-term contingency [7–10]. Indeed, there is evidence of reciprocal exchanges of various kinds in over 160 species [10]—some species may follow simple decision rules such as ‘help anyone if helped by someone’ (i.e. ‘generalized reciprocity’) and others may apply more cognitively demanding ones, such as ‘help someone who has helped you before’ (i.e. ‘direct reciprocity’). In this regard, short-term, direct reciprocity could be particularly powerful for promoting and stabilizing cooperation in humans [11], but it is controversial whether it also applies among our nearest primate relatives, the great apes, and particularly when food is involved.

There is much evidence that great apes engage in reciprocal activities such as grooming, coalitional support, infant handling and play [12–19]. Such activities of reciprocal help cultivate cooperative partners (i.e. ‘friends’)—in both affiliative and competitive social relationships [9,20–23]. However, in-kind food exchanges, such as ‘food-for-food’, are rare among great apes [16,24,25]. One possible explanation for this could be that great apes have evolved to view food as a target of competition, determined by dominance, not as a commodity for social exchange and friend-making [26,27]. Another explanation could be that, unlike reciprocating grooming or consolation, delivering food entails an immediate material loss [20]. Some support for these possibilities could be that in social groups with relatively shallow dominance hierarchies, food is distributed more evenly among members, and the way of distribution is primarily tolerance to scrounging rather than pro-active sharing [28]. Studying great apes in the wild offer meaningful associations between food that is given and food that is received, yet it is debatable whether food exchanges in great apes is based on contingent reciprocity [29], coercion [30] or a mixture of the two [24]. Notably, an inherent challenge with natural observations is controlling for confounding factors such as spatial proximity, social rank and kin-relations [17,31,32]—all of which hinder the ability to conclude whether observed reciprocal behaviour is indeed an independent capability.

A recent review of reciprocal help in non-human species covered about 130 observational and experimental studies and noted several differences that may contribute to the current gap regarding reciprocity in chimpanzees [20]. The first difference is in methodological weaknesses such as the inability to infer causation from correlational evidence in observational settings and the challenge of ensuring task understanding while avoiding over-training in experimental settings. A second difference is the usage of different exchange commodities. Specifically, reciprocal exchanges in the wild are far more likely to involve grooming, support, tolerance, aggression, mating, play and infant handling and are less likely to involve food—which is the common commodity used in experimental studies. A third difference is the impact of relational bonds, as 80% of natural observations investigated interactions among kin-related individuals, and 90% of experimental studies investigated non-kin, thus suggesting that reciprocity in the wild could be confounded with kinship. Lastly, a fourth difference regards temporal consideration. Specifically, as affiliative stances motivate reciprocal behaviour (e.g. sympathy and resentment) [33], natural observations address social interactions in which positive and negative relational bonds have already been established through time [34]. By contrast, experimental procedures implement quick and short interactions, which may not have sufficient power to elicit affiliative stances between conspecifics, arguably crucial for reciprocal behaviour.

A relevant recent empirical demonstration of exchanging ‘favours’ in chimpanzees was provided by Schmelz *et al*. [35]. Two chimpanzees were positioned in separate cages, and one chimpanzee could choose between two options: (i) a ‘safe’ distributive option that guaranteed a food reward for herself (but guaranteed nothing for the second chimpanzee), or (ii) a ‘risky’ option which passed the decision to the second chimpanzee. If the first chimpanzee chose the ‘risky’ option, the second chimpanzee then had a choice between another two options: (i) delivering food only to herself or (ii) delivering food to the first chimpanzee as well. The findings suggest that in response to a ‘risky’ choice taken by the first chimpanzee, the second chimpanzee was more likely to provide food for her in return (note that the experiment was rigged so that this choice only *appeared* to be ‘risky’ from the perspective of the second chimpanzee). Interestingly, follow-up studies found that chimpanzees did not act to provide food to others that they had observed providing benefits to third parties [36], suggesting that the psychological mechanism at work is not for finding individuals who are good cooperators but rather for friendship formation via direct reciprocity of different commodities. This finding implies a reciprocal exchange of ‘favours’ such as ‘food-for-instrumental help’, but we have no way of knowing how differences in the value of these different currencies impact this reciprocity. ‘Food-for-food’ exchanges would avoid this issue by using exchanges of items in the same currency, and it would inform our understanding of the origins of food exchanges in humans.

‘Food-for-food’ exchanges in chimpanzees have been examined in the ‘prosocial task’ used by Silk *et al*. [37] and Brosnan *et al*. [38] (also used by Schmelz *et al*. [35] for one-way food transfers). In this paradigm, one chimpanzee was allowed to choose between providing one piece of food for both herself and a conspecific (i.e. ‘1–1’; ‘prosocial’ option) or – providing one piece of food for herself and nothing for the conspecific (i.e. ‘1–0’; ‘selfish’ option). In these studies, chimpanzees did not demonstrate spontaneous prosocial tendencies at all [37] and only 1 of 11 dyads demonstrated contingent reciprocal prosocial behaviour [38]. One reason for this result could be that great apes have evolved to view food as a target of competition, determined by dominance, not as a commodity for social exchange and friend-making [26,27]. Thus, it is not surprising that experimental studies demonstrated exchanges of different kinds of ‘favours’ [35,39,40] but not in-kind spontaneous food exchanges [24,38,41,42]. Other reasons could be limitations of experimental methods, such as the tension between ensuring task understanding while, at the same time, avoiding over-training subjects. In this regard, about 75% of experimental studies that have implemented the ‘prosocial’ task did not sufficiently demonstrate task understanding [43]—thus hindering the ability to conclude whether an absence of evidence reflects an absence of the phenomena or instead reflects the challenge of designing adequate methods that can expose reciprocal behaviour persuasively.

Here, we bridge observational and experimental approaches and address two questions regarding food exchanges by testing chimpanzees, a few bonobos, and young children. The first question focuses on positive reciprocal food exchanges (i.e. ‘food-for-food’): what will individuals do in the prosocial task when a conspecific ‘intentionally’ gives them food when they *could* have given nothing (i.e. the experiment was rigged so that the conspecific appeared to deliberately choose ‘1–1’ instead of ‘1–0’). If chimpanzees will reciprocate in kind towards the benefactor, it would suggest that the evolutionary roots of reciprocal food exchange in humans probably involved some diminution of dominance and aggression around food so that the processes of reciprocity at work in other domains could work with food as well (as suggested to be the case in bonobos' lesser aggressiveness and greater spontaneous food sharing in an experimental setting [44]).

While the mechanism of positive reciprocity can *foster* cooperation, our second question asks whether non-human great apes also exhibit negative reciprocity, a mechanism that can arguably *stabilize* cooperation [45]. Specifically, we used negative reciprocal food exchanges (i.e. ‘no-food for no-food’) to ask what chimpanzees would do when another individual ‘*intentionally*’ chose *not* to give them food (i.e. the experiment was rigged so that the conspecific appeared to deliberately choose ‘1–0’ instead of ‘1–1’). If chimpanzees reciprocate in kind toward a malefactor, this could provide evidence for a behaviour thought to be more common in humans than other great apes, namely punitive behaviour to deter ‘bad actors’ [42,46].

**Figure 1.**
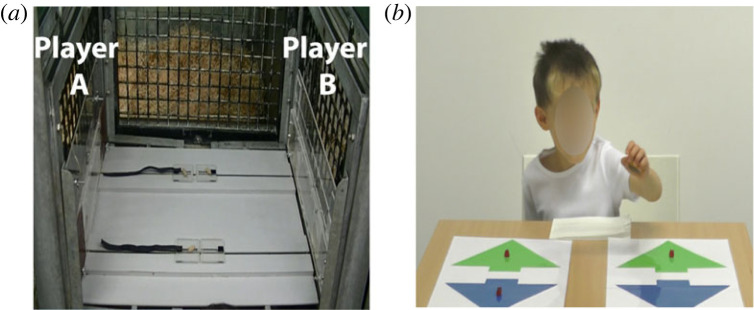
Prosocial game for great apes and children. (*a*) For great apes, one apparatus was used in the *preliminary*, *test* and *post-hoc* phases. Great apes were positioned as ‘player A’ in the *preliminary* phase, and as ‘player B’ in the *test* and *post-hoc* phases. Rewards were placed in alternating sides per trial (upper and lower), thus – if subjects were completely indifferent and simply choose the option that was closest to them, then no significant preference will be found (i.e. 50–50). (*b*) For young children, gender and age-matched unfamiliar partners were presented via pre-edited videos. The procedure for children included 2 rounds in which a partner makes a deliberate, explicit and consistent distributive choice (i.e. always egalitarian, or always selfish), and only in the third round were participants allowed to respond. Valued edible rewards were peanuts for chimpanzees, grapes for bonobos and gummy bears and candies for children.

We created an experimental procedure comparable to prior work that *did not* find evidence for reciprocal food exchanges [38]: the prosocial task, using food as the exchanged commodity and unrelated subjects (figure 1*a*). However, our procedure differs from prior studies by including several features necessary to elicit reciprocal behaviour within a short-term experimental interaction [9,47]. We did this by manipulating the experimental interaction between two conspecifics, so that (i) the distributive choices made by the first chimpanzee (player ‘A’ in figure 1*a*) were consistently prosocial (i.e. 1–1) or consistently selfish (1–0) for two consecutive rounds, and (ii) it appeared to the second chimpanzee (player ‘B’ in figure 1*a*) that player ‘A’ had made *deliberate* choices. We hypothesized that embedding these design features within a short-term experimental setting would be more likely to elicit an attitudinal stance toward the benefactor/malefactor than did prior experimental studies, which did not include these features that are crucial for the reciprocal act [33]. The procedure for the great apes included three phases—*preliminary*, *test* and *post hoc* phases (see electronic supplementary material, table S1 for a detailed list of all conditions and electronic supplementary material, videos S1 and S2 for examples from the test phase). The procedure with 4-years-old children was more straightforward and used the same task (i.e. ‘1–1’ versus ‘1–0’, with candies as rewards) as to allow comparing the rate of reciprocal food exchanges in each species. Previous findings with this task demonstrated no initial pro-social preferences at these ages [48].

**Figure 2.**
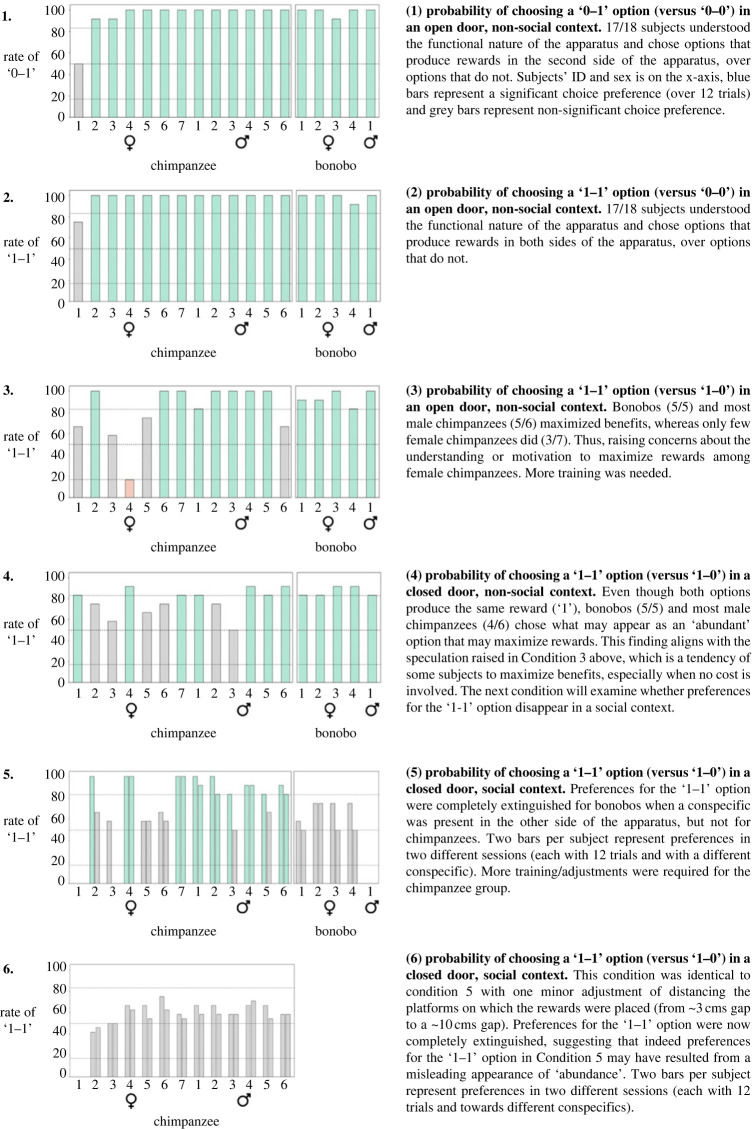
Six conditions of the *preliminary* phase. A 2–3-week gap was maintained between conditions and sessions. Blue bars represent a significant preference for the ‘1–1’ option (i.e. at least 10/12 trials); grey bars represent non-significant preference. Note: subject no. 1, who failed in most conditions, was used as a stooge during the *test* phase (see Methods Summary).

The *preliminary* phase was designed to clearly demonstrate that the great apes understood the apparatus before moving on to the test trials. The phase included six conditions (each consisting of 12 trials) in which subjects played as active distributors (player ‘A’ in figure 1*a*). The findings of this phase were that, by the time the *test* phase began, (1) we knew that subjects are motivated to obtain the pieces of food, (2) subjects understood the mechanism of food delivery, (3) subjects understood that only one distributive choice could be made in each trial, and (most importantly) (4) none of the subjects had egalitarian or selfish preferences toward conspecifics, a finding that replicates prior work [37] (figure 2; and in more depth in electronic supplementary material, figures S1–S6).

**Figure 3.**
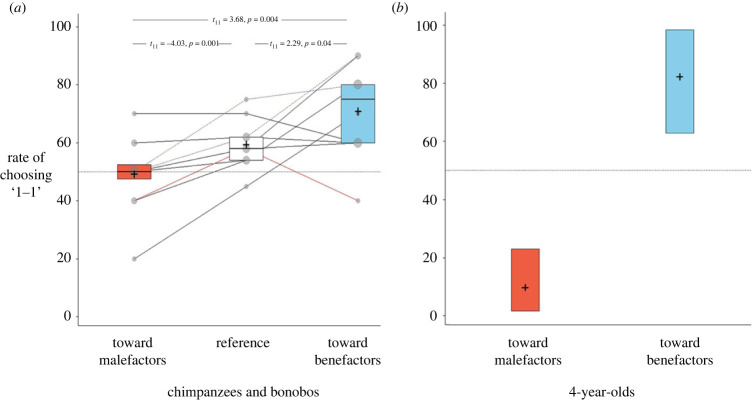
Rate of prosocial choices (1–1). (*a*) Rate of prosocial choices among chimpanzees (*n* = 10; solid black lines; red line represents the chimpanzee alpha male) and bonobos (*n* = 2; dotted lines) towards prior malefactors and benefactors (*test* phase) compared to their reference achieved at the end of the *preliminary* phase (condition 5 for bonobos and condition 6 for chimpanzees). (*b*) Rate of prosocial choices among 4-year-olds. Note: since great apes were tested in a within-participant design and children in a between-participant design, the datasets cannot be compared directly.

By playing the role of player ‘A’ during the *preliminary* phase, subjects experienced having a ‘free choice’ between the two distributive options. However, in the *test* phase—subjects were assigned to a role they had never played before, i.e. as recipients (player ‘B’). Now, the apparatus was rigged such that the partner (i.e. player ‘A’) could only make egalitarian choices (i.e. ‘1–1’; positive reciprocal food exchange condition) OR only selfish ones (i.e. ‘1–0’; negative reciprocal food exchange). Rigging the apparatus allowed us to ensure that partners act in a consistent manner towards the subject (i.e. now being player ‘B’) for two consecutive rounds (see electronic supplementary material, videos S1 and S2). If subjects perceived the partner's behaviour as deliberate and consistent, they might be more likely to develop a positive attitude toward an egalitarian partner and a negative attitude toward a selfish partner. Following a partner's consistent behaviour toward a subject for two rounds, the subject was then allowed to respond spontaneously with the same distributive options. The main finding of the *test* phase was that in the negative reciprocal food exchange condition, subjects made significantly fewer ‘prosocial’ choices than their reference tendency to make prosocial choices at the end of the *preliminary phase* (*t*_(11)_ = −4.03; *p* = 0.002). Conversely, subjects made significantly more ‘prosocial’ choices in the positive reciprocal food exchange condition than their reference tendency (*t*_(11)_ = 2.29; *p* = 0.04). Together, these findings provide an experimental demonstration of both *negative* and *positive* reciprocal food exchanges in great apes (figure 3*a*). In young children, we found greater extents of positive and negative reciprocal food exchanges (figure 3*b*), and the implications will be further discussed.

## 2. Results

For the great apes, we analysed the data with a linear model (lmer), which included the rate of choosing the prosocial choice as a dependent variable (i.e. rate of ‘1–1’ choices across ten trials), and as predictors reciprocal condition (positive, negative and reference as a dummy variable), sex, the interaction between them, the order of the reciprocal conditions (positive first or positive second) and subject ID. We did not have a specific hypothesis about sex but included it since males and females seemed to behave slightly different in some conditions during the preliminary phase (i.e. conditions 3 and 4 in figure 2). Inspection of model assumptions revealed no problems regarding collinearity (largest variance inflation factor = 1.03), no influential cases (DFFits max value = 1.2), nor problems with model stability (i.e. Cook's distance = 0.23, leverage = 0.16). Two points should be noted: First, the behaviour of the chimpanzee alpha male in the positive reciprocity condition was exceptional yet not exceeded the threshold for being an outlier (for exact numbers see comments in the R code file at https://osf.io/r2k84). We took a conservative approach and included him in the model nonetheless. Excluding the alpha male does not change the results (see electronic supplementary material, figure S8A). Second, we did not include ‘species’ as a factor due to the small number of bonobos (*n* = 2). Excluding bonobos does not change the results (see electronic supplementary material, figure S8B) and bonobos' behaviours did not influence the model, in other words, they were not ‘outliers’ (for exact numbers see the R code file as noted above).

Likelihood ratio test comparison between the full and null models was highly significant ($\chi_{9}^{2}=19.46$, *p* = 0.002). A 2-way interaction between reciprocal condition and sex was not significant ($\chi_{9}^{2}=2.25$, *p* = 0.32), and a main effect was found for reciprocal condition ($\chi_{2}^{2}=17.1$, *p* < 0.001), which was then followed by two two-tailed paired *t*-tests. Indeed, in the negative reciprocity condition, subjects were less likely to make ‘prosocial’ choices than their reference tendency (*t*_(11)_ = −4.03; *p* = 0.002), and in the positive reciprocity condition, subjects were more likely to make ‘prosocial’ choices than their reference tendency (*t*_(11)_ = 2.29; *p* = 0.04).

For young children, given the between-participants design, we analysed the data with a general linear model (glm), which included their choice as a dependent variable (i.e. ‘1–1’ or ‘1–0’; prosocial or selfish), and as predictors reciprocal condition (positive or negative), gender, the interaction between them and age in months as a covariate. Likelihood ratio test comparison between the full and null models was highly significant (*p* < 0.001) and revealed only one main effect for reciprocal condition (estimate ± s.e. = 5.86 ± 1.69, *z* = 3.48, *p* < 0.001), suggesting more ‘prosocial’ choices toward benefactors than malefactors. Two binomial tests per condition further revealed strong prosocial preferences toward benefactors (approx. 80%; 20/24; *p* = 0.001), and strong selfish preferences toward malefactors (approx. 90%; 22/24; *p* < 0.001).

### (a) Alternative explanations for reciprocal behaviour of great apes and children

An alternative explanation for the reciprocal food exchanges in great apes discovered above could be a potential bias of the great apes to follow and choose the option of the *same side* their partner previously chose (i.e. ‘local enhancement’). A *post-hoc* phase was designed to address this concern and was conducted three months after all subjects had completed the *test* phase to allow the impact of the *test* phase to fade. The *post-hoc* phase included two control conditions. Each included ten trials and a 2–3-week gap in-between conditions. In the first control condition (non-rewarding local enhancement), player ‘A’ chose one of two non-rewarding distributive options (i.e. ‘1–0’ or ‘1–0’) from the same side for two consecutive rounds, and then the subject (player ‘B’) was allowed to respond by choosing between two identical options (i.e. ‘1–1’ or ‘1–1’.). If subjects were found to be biased to choose the same side that the partner chose, then our conclusion from the *test* phase about negative reciprocity will be rejected. The findings revealed that only 1 of 10 subjects consistently chose the same side as their partner in the ‘non-rewarding’ condition. In the second, more dramatic control condition (‘rewarding local enhancement’ condition), a partner chose one of two rewarding distributive options (i.e. ‘1–1’ or ‘1–1’) from the same side for two consecutive rounds, and then subjects were allowed to respond by choosing between two identical options (i.e. ‘1–1’ or ‘1–1’). In this condition, if subjects were biased to choose the side from which the partner had rewarded them, then our conclusion from the *test* phase about positive reciprocity will be rejected entirely. The findings were that 0 of 10 subjects chose the same side as their partner, suggesting that local enhancement was not a force driving chimpanzees' behaviour in this task (see electronic supplementary material, figure S9). A notable behaviour that should be mentioned was that of the chimpanzee alpha male, who was the only one who demonstrated a significant preference to choose the *same* side as the partner in the ‘non-rewarding’ control condition, and a near significant preference to choose the *opposite* side from the partner in the ‘rewarding’ condition (see electronic supplementary material, figure S9), thus highlighting the possibility for ‘human-like’ reciprocal food exchanges in the diminution of clear dominance relations.

An alternative explanation can also be directed at the high rates of positive and negative reciprocity in children (i.e. approx. 80% and approx. 90%, respectively). Since the interactions between partners and child participants involved verbal communication (i.e. ‘one for me, and one/zero for you’), it is at least possible that such statements may intensify children's in-kind responses. However, recent findings suggest otherwise [49–51]. One closely related example would be the study of Zhang *et al*. [50], who examined the influence of intentions and outcomes on reciprocal sharing of 3- to 5-year-old children (from the same background as in our study). In some conditions partners explicitly communicated good intentions (i.e. ‘I really like these stickers, but you know what, I will give you three stickers'), and in other conditions communicated bad intentions (i.e. ‘I like these stickers very much, and I will not give you any; I will keep them all’). What was found is that if the outcome of the partner's behaviour was ‘bad’ (i.e. no stickers were delivered), then children responded selfishly whether partners had ‘good’ or ‘bad’ intentions, in other words—when the outcome was bad, the intentions did not matter. Even more so, in conditions with ‘good intentions and good outcomes', Zhang *et al*. [50] report that 75% of 5-year-olds reciprocated spontaneously without being any need verbal cues or requests from partners, which is similar to the approximately 80% rate in our positive reciprocity condition. Complementary, in conditions with ‘bad intentions and bad outcomes’, 85% did not share anything spontaneously and only upon receiving verbal cues or requests from partners, which is similar to the approximately 90% rate in our negative reciprocity condition. Together, the possibility that verbal communication between partners and children may have increased the rates of reciprocal behaviour, and thus incomparable to great apes, seems unlikely.

## 3. Discussion

By designing a rigorous experimental setting that incorporates social cues that elicit reciprocity in natural dyadic interactions [47], this study finds clear evidence for reciprocal ‘food-for-food’ exchanges in non-human apes. Consistent with prior studies, our *preliminary* phase shows no spontaneous prosocial preferences among great apes in the ‘prosocial’ task. That is, when apes were allowed to *initiate* a prosocial act towards conspecifics, the likelihood of prosocial behaviour was 58% in our sample (figure 2) and 58% in Silk *et al*. [37], supporting their finding that chimpanzees were ‘indifferent’ and showed no evidence for other-regarding preferences with this task. However, in our *test* phase, apes were allowed to *respond* to a conspecific who had previously appeared to demonstrate a *deliberate* and *consistent* behaviour that yields a ‘desirable’ or an ‘undesirable’ outcome for them. In these cases, great apes engaged in direct reciprocal food exchanges—both positively and negatively. Importantly, this direct reciprocal behaviour did not stem from methodological confounds such as ‘local enhancement’ (i.e. *post-hoc* phase), suggesting that it is genuinely contingent prosocial behaviour. Notably, bonobos behaved similarly to the chimpanzees (figure 3) and were not found to be outliers in the preliminary analysis. Nonetheless, given that only two bonobos participated in the test phases, we should be cautious making general conclusions about them.

Our procedure allowed us to address, although indirectly, possible explanations for why food exchange are less common in the wild compared to exchange of other commodities [10,20]. One explanation would be that obtaining food is never guaranteed in competitive social environments, thus food likely is to be viewed as a commodity to compete for, rather than to trade with. Conversely, food is provided on a regular basis for captive chimpanzees. Food is thus more likely to be viewed as an abundant resource, and this should increase the possibility of being tradable. Relatedly, another explanation would be that unlike reciprocal grooming, sharing food with another involves an immediate material loss. In our procedure, the possibility of ‘loss aversion’ was reduced since subjects were rewarded regardless of their distributive choice (i.e. ‘1–1’ or ‘1–0’). Together, it is at least possible to assume that in addition to the intentional and consistent behavioural signals of partners in the ‘positive’ and ‘negative’ reciprocal conditions, the confidence of being provided with food regularly, and the reduction of ‘loss aversion’ in our task—may have reduced motivations to obtain and withhold food, and allowed us to expose social interactions that were not selfish nor altruistic, but rather reciprocal. On this basis, future investigations should further explore other factors that may facilitate direct reciprocity in great apes, such as using payoff-matrices that are more costly [48], manipulating rank differences within dyads [34], or even offering several reciprocal options to choose from (as suggested by Schweinfurth & Call [20]). Such systematic investigations could inform us about the conditions under which trade can flourish and also about the frequencies of different forms of trade, i.e. between different commodities (e.g. food for help), similar commodities (e.g. grooming for grooming), and the specificity of trade with similar commodities (e.g. in-kind grooming exchange versus in-kind food exchange) [10,20].

Here, our questions were directed to explore whether the uniquely human propensity for in-kind food exchanges is more-likely due to derived changes in the cognition of reciprocity, or changes in tolerance/competitiveness for food. Our first question was about whether we can demonstrate positive and negative in-kind reciprocal food exchanges among chimpanzees (and few bonobos). Moreover, understanding the similarities and differences between great apes' and humans’ willingness to exchange food would inform our models regarding the origins of uniquely human forms of cooperation. Regarding positive reciprocity, we found that in-kind food exchanges are not only possible in great apes, but they reach the same level as in human children (approx. 75–80%; figure 3). This suggests that under naturalistic conditions, positive reciprocity in food exchanges is rare in non-human apes due to interference by some other social phenomenon or dynamic, such as dominance. Though only speculative, this account seems consistent with our finding that the chimpanzee alpha male behaved differently from all the others.

Our second question concerned negative reciprocal food exchanges. In the negative reciprocity condition, apes' behaviour could be interpreted as ‘random’ when looking at the group average (i.e. approx. 50% of ‘prosocial’ choices), yet almost all subjects adjusted their behaviour to be less prosocial towards malefactors than their personal baseline. Here, a big gap was found in the rates of negative reciprocal food exchanges among great apes (i.e. approx. 50%(and young children (i.e. approx. 90%), which could be interpreted to mean that punitive actions toward selfish partners might ‘matter’ more to young children than they do to chimpanzees. We know that great apes are motivated by self-oriented considerations in laboratory experiments [42]. Thus, in our studies, chimpanzees perhaps believed they could maximize their future gains by positively reciprocating prosocial behaviour—but not by reciprocating non-prosocial behaviour (i.e. acting punitively). This suggests that punitive actions may not be as strong a motivator of reciprocal cooperation in great apes, at least in these laboratory experiments. Such an explanation alludes to the role of reciprocal mechanisms in facilitating cooperation via *promoting* interactions with ‘good’ actors (e.g. partner choice [52–55]) on the one hand and via *deterring* ‘bad’ actors on the other hand. Our findings suggest that the former is shared among great apes and human children, but the latter is found primarily among human children and could be an essential piece in the evolutionary story regarding the function of punitive sanctions to stabilize group-level cooperation [11,46,56]. Broadly speaking, as humans have evolved specialized skills of social cognition [57], our findings suggest that the quantitative difference in negative reciprocity between great apes and young children could reflect a broader qualitative difference, which was found to support human children to both form *and* sustain group-level ‘culture’ [58].

## 4. Materials and methods

### (a) Children

#### (i) Sample

We tested 48 4-year-olds (*M*_age_ = 4.6, s.d. = 0.1, 50% girls) paired with gender and age-matched unfamiliar partner (via pre-edited videos). Four-year-olds were the youngest age who were able to undergo the procedure without special assistance from the experimenter (e.g. counting the rewards, and understanding the directionality of the arrows). To maintain a narrow age range of 4.3–4.8, the sample was recruited from 10 kindergartens in Leipzig, Germany, and all had parental consent to participate.

#### (ii) Design

A between-participants factorial design included partner's behaviour (varies as *cooperative* or *selfish*; ‘1–1’ or ‘1–0’, respectively) as an independent variable and participant choice as the dependent variable (varies as *cooperative* or *selfish*). Power analysis using pwr.f2.test (‘pwr’ package v. 1.3–0 in R) included three factors (i.e. gender, partner's behaviour and an interaction), a sample of 48 participants, and an estimated medium effect size (*f*^2^ of 0.25), and showed that the probability for detecting actual effect is 86%.

#### (iii) Materials

To maintain high levels of engagement, we used different types of edible rewards for each of the three distributive rounds (e.g. gummy bears and sweets).

#### (iv) Partners

Partners were age and gender-matched and unfamiliar to the participants. Partners were presented via pre-edited videos in a lively manner for about 30 s. The videos were designed to present a partner who makes deliberate, explicit, and consistent distributive choices toward the participant. Specifically, the partner faced the camera (i.e. looking at the participant), then looked back and forth at the distributive options laid in front of him/her, as if considering the options. The partner looked up at the camera again, pointed at the distributive option, and explicitly verbalized it. For example, in a cooperative condition, the partner pointed to the desired choice and stated, ‘I choose this!’ followed by ‘One for me {point to oneself} and one for you {point to the camera}!’ whereas, in a selfish condition, the partner stated ‘One for me [point to oneself] and zero for you [point to the camera]*!*’.

#### (v) Procedure

One experimenter, blinded to the research hypothesis, sat individually with every child in a quiet room in their kindergarten. The experimenter opened with, ‘In this game, you can win gummy bears and sweets, but first, let me explain how the game works'. A short warm-up phase was done to introduce the game and ensure that children understood the directionality of the rewards and that they could count the number of rewards correctly. In this warm-up phase, we used small plastic discs as rewards, the distributive options were 2–2/2–0, and no partner was presented. Thus, participants were unfamiliar with the rewards, the distributive options, and the partners used in the experimental conditions. The experimenter then introduced the game and stated, ‘Now you will play with another child, the other child will play first, then it will be your turn, and that is it. Let us see whom you will play with’. The experimenter played the first video and said, ‘This is Dan/Dana. What type of rewards are on the board?’ (Gummy bears, sweets; all children identified the rewards correctly). Following the partner's choice (1–1/1–0), the experimenter asked, ‘So, how many gummy bears did Dan/Dana choose for himself/herself? … and how many did you get?’. All children answered correctly, and the experimenter placed the appropriate number of rewards in an envelope with the child's name. Children continued to play as passive recipients for one more round, with different rewards (i.e. gummy bears, sweets). Finally, a third round was played in which children played as active distributors. Specifically, the experimenter said, ‘OK, so now it is your turn to choose!’, then placed gummy bears in front of the child, played the third video, and wondered, ‘let us see whom you are going to play with … oh, it is Dan/Dana’. Children made their choice, and the game was over.

### (b) Great apes

#### (i) Sample

The *preliminary* phase included 18 subjects—13 chimpanzees (*M*_age_ = 25.5, s.d. = 0.5, 54% females) and 5 bonobos (*M*_age_ = 14.7, s.d. = 4.5, 80% females)—all housed at the Wolfgang Kohler Primate Research Center in Leipzig, Germany. The *test* phase included 12 subjects—10 chimpanzees and 2 bonobos—for reasons that will be explained shortly, and the *post-hoc* phase was done with 10 chimpanzees. All available subjects participated in the *preliminary* phase, but we had to exclude a few from participating in the *test* and *post-hoc* phases for two main reasons. First, the *test* phase required a ‘stooge’ for the role of player ‘A’. The ‘stooge’ becomes aware that he/she does not have a ‘free choice’ between egalitarian or selfish distributive options since the apparatus is rigged. Since the *test* phase paired sex-matched dyads, we thus had to choose 3 subjects that would not participate in the *test* phase – one female chimpanzee (*Corrie*, who had also failed most conditions in the *preliminary* phase), one male chimpanzee (*Bangolo*, the youngest), and one female bonobo (*Fimi*), and assigning them as stooges (i.e. the role of player ‘A’). In addition, one male bonobo participated in the *preliminary* phase but could not be paired with another male for the *test* phase since there was none. Second, one female chimpanzee and one female bonobo were unmotivated to participate in the *test* phase (*Fraukje* and *Yasa*, respectively). Notably, all chimpanzee subjects who participated in the *test* phase participated in the *post-hoc* phase and with the *same partners* as in the *test* phase.

#### (ii) Design

A round-robin design was implemented (see electronic supplementary material, table S2). To illustrate, once all subjects completed the *preliminary* phase, one female chimpanzee was chosen to play as a ‘stooge’ in the *test* phase (i.e. *Corrie* as player ‘A’). After interacting positively and negatively with another female (e.g. *Riet*, being player ‘B’), we now doubled the number of available ‘stooges’ and could assign either *Corrie* or *Riet* as ‘stooges’ to interact with other females. The same procedure was done with male chimpanzees and female bonobo. Overall, the first subjects in each species and sex group interacted with the same stooge (positively and negatively with a 2–3-weeks gap in between), and other subjects interacted with two different stooges (a benefactor and a malefactor, with a 2–3-week gap in between), and in counterbalanced order (i.e. benefactor first or second). The order of interacting with a benefactor or a malefactor had no impact on subjects' behaviour, and looking separately at those who interacted with a benefactor first (versus benefactor second) follows the behavioural pattern as in figure 3*a* (see electronic supplementary material, figure S7).

#### (iii) Materials

We used one apparatus for all phases (*preliminary*, *test*, and *post-hoc*), representing the standard setup others have used to assess prosociality [37]. Subjects had the option of pulling one of two short Velcro stripes (figure 1). One of the stripes triggered a selfish resource distribution (1–0; one reward for the subject and zero for the partner), and the second stripe triggered a prosocial resource distribution (1–1; one reward for the subject and one for the partner). To prevent individuals from choosing both options simultaneously, pulling a stripe was possible by first sliding a Plexiglas door that allowed access to the desired Velcro stripe. Once the Plexiglas door had been moved to either side, it was automatically locked and could not be moved to allow further access to the remaining choice. E had released the locked Plexiglas door before each trial (see electronic supplementary material, videos S1 and S2). As rewards, peanuts were used for chimpanzees and grapes for bonobos.

## Data Availability

The data and code were uploaded to: https://osf.io/uzn7w/?view_only=aeb681217f8e484aa0693cdb2e468c73. The data are provided in electronic supplementary material [59].
